# Getting Grip on Phosphorus: Potential of Microalgae as a Vehicle for Sustainable Usage of This Macronutrient

**DOI:** 10.3390/plants13131834

**Published:** 2024-07-03

**Authors:** Alexei Solovchenko, Maxence Plouviez, Inna Khozin-Goldberg

**Affiliations:** 1 Department of Bioengineering, Faculty of Biology, Lomonosov Moscow State University, 1-12 Leninskie Gory, 119234 Moscow, Russia; 2 The Cawthron Institute, Nelson 7010, New Zealand; maxence.plouviez@cawthron.org.nz; 3 Microalgal Biotechnology Laboratory, French Associates Institute for Agriculture and Biotechnology of Drylands, Ben-Gurion University of the Negev, Sde-Boqer Campus, Midreshet Ben-Gurion 8499000, Israel; khozin@bgu.ac.il

**Keywords:** polyPhosphate(s), luxury phosphorus uptake, wastewater, biofertilizer, phycoremediation

## Abstract

Phosphorus (P) is an important and irreplaceable macronutrient. It is central to energy and information storage and exchange in living cells. P is an element with a “broken geochemical cycle” since it lacks abundant volatile compounds capable of closing the P cycle. P fertilizers are critical for global food security, but the reserves of minable P are scarce and non-evenly distributed between countries of the world. Accordingly, the risks of global crisis due to limited access to P reserves are expected to be graver than those entailed by competition for fossil hydrocarbons. Paradoxically, despite the scarcity and value of P reserves, its usage is extremely inefficient: the current waste rate reaches 80% giving rise to a plethora of unwanted consequences such as eutrophication leading to harmful algal blooms. Microalgal biotechnology is a promising solution to tackle this challenge. The proposed review briefly presents the relevant aspects of microalgal P metabolism such as cell P reserve composition and turnover, and the regulation of P uptake kinetics for maximization of P uptake efficiency with a focus on novel knowledge. The multifaceted role of polyPhosphates, the largest cell depot for P, is discussed with emphasis on the P toxicity mediated by short-chain polyPhosphates. Opportunities and hurdles of P bioremoval via P uptake from waste streams with microalgal cultures, either suspended or immobilized, are discussed. Possible avenues of P-rich microalgal biomass such as biofertilizer production or extraction of valuable polyPhosphates and other bioproducts are considered. The review concludes with a comprehensive assessment of the current potential of microalgal biotechnology for ensuring the sustainable usage of phosphorus.

## 1. Introduction: Peculiarities of Phosphorus as a Macronutrient

No living cell, including microalgal cells, can exist without phosphorus (P). This nutrient plays a central role in the storage and transduction of energy and information therein and serves as an important structural component of the cell. The main organic P pools of the cell include nucleic acids, phospholipids, and diverse low molecular weight phosphometabolites. The main inorganic P pools are dissolved inorganic phosphate, orthophosphate (P*_i_*), and its homopolymer polyPhosphate (PolyP, see Section 1.2.4), the main reserve form of P in the cell (the roles of P and its main pools in the cells are briefly discussed in Section 1.2.1 and Section 1.2.2). P*_i_* is itself a key metabolite existing in a delicate equilibrium with a broad array of P metabolites of central metabolism including Calvin cycle, turnover of sugars, adenylates, and nucleotides [1]. 

Unlike the major biogenic elements such as O, N, or C; P slowly enters the biosphere, mainly during the weathering of the base rock releasing P*_i_* into the pedosphere and hydrosphere. There, due to its high reactivity, a significant part of P becomes bound into poorly soluble compounds [2]. Therefore, P is characterized by a low bioavailability despite its high abundance (P is the 11th most abundant element in the Earth’s crust [3], see also Section 1.1). Some particulate P*_i_*-containing material is deposited on ocean and lake bottoms, where it can be recycled at an extremely slow rate. The net result of this is a very slow turnover of P which is even called “an element with a broken geochemical cycle” [4] and living organisms most of the time experience a shortage of P and therefore become adapted to it in the process of evolution [5].

Recently, humankind exerted a profound impact on P cycling in the biosphere. In the pre-industrial epoch, the production of food depended mostly on and was limited by the availability of organic waste as a source of P. With the intensification of crop production after the green revolution, the demand for P fertilizer soared and extensive mining of rock phosphates for fertilizer production created two major concerns [6,7,8]: dwindling of P resources and wasteful P usage. Recently estimated rock phosphate global consumption exceeds 160 million tons or ca 21 million tons of P [9]. These “concentrates” or, in other words, economically extractable sources of P may be exhausted in the near future, although what is “economically extractable” will certainly change with increasing demand for P and technological development. This will lead to an unprecedented crisis in agriculture complicated by the extremely uneven distribution of the minable rock phosphate between different countries similar to the distribution of oil and other fossil hydrocarbons [4,8]. Nevertheless, there is a crucial difference between fossil fuel and P: while the former can be replaced with alternative carbon and energy sources, P is irreplaceable. Although new deposits may be found (e.g., Norway), solving the broken P cycle is still of critical importance for global agriculture.

Furthermore, the usage of P fertilizers is woefully inefficient: in many cases, less than 20% of P applied with fertilizers is taken up by crop plants [10]. These losses along with those in the supply chain of agricultural products are leaching from agricultural soils to terrestrial and aquatic ecosystems. The abrupt increase in P*_i_* availability promotes algal and cyanobacterial blooms [11] with subsequent depletion of O_2_ in the environment and suffocation of the hydrobionts [12]. Frequently, eutrophication becomes exacerbated by the proliferation of toxic cyanobacteria and dinoflagellates whose toxins increase the damage to the ecosystems [13].

While society becomes increasingly aware of the non-renewable and finite nature of P resources (see Section 1.1), the paramount challenge of establishing a sustainable anthropogenic phosphate cycle has not changed in the last decade [6]. The need to act is urgent and several action priorities have been recently suggested to cope with the P shortage, including recovery of phosphate from anthropogenic point sources, precision fertilizer placement technology, engineering of plants for minimizing their P requirements, and maximizing phosphate uptake [7,14,15]. The evidence from the fields of microalgal physiology, ecology, and biotechnology strongly supports the idea that at least some of these priorities are attainable with the use of microalgal cultures. 

Under abundant P availability conditions, microalgal cells take up much more P than is necessary for the next cell division judging from the Redfield ratio 106:16:1 (C:N:P) [16]. Previous estimation of the global potential of non-optimized P uptake capacity of cultivated microalgae [7] (calculated on a rather optimistic assumption of 40 g (DW) ∙ m^–2^ ∙ day^–1^ and a modest cell dry weight P percentage of 1.8%) suggests that a production area of approximately 26,600 km^2^ (a little more than, e.g., the area of Israel) would be required to sequester the 7 Mt of P that is annually lost worldwide with animal manure. In reality, the required area could even be less since microalgal P uptake and accumulation capacity can be boosted to at least 7% of Cell DW [14,15]. Therefore, the goal of increasing the sustainability of using P with the help of microalgal biotechnologies seems to be, in principle, feasible. Moreover, the potential of microalgal biotechnology for P upcycling is vigorously discussed in the recent literature (the number of publications retrieved by a Scopus query “microalgae phosphorus removal” increased more than three times over the last 10 years, see also [17,18]). At the same time, there are limitations and concerns (which are often overlooked) that should be considered and circumvented before the practical implementation of the microalgae-based technologies for P biocapture. Towards this end, we attempted to summarize a broad spectrum of opinions from somewhat pessimistic to strongly optimistic about the potential of microalgal biotechnology for achieving the sustainable usage of P, particularly in agricultural systems, and in the frame of the development of innovative bio-economies.

### 1.1. Global Perspective on P: A Nutrient That Is “Abundant but Scarce” and Mostly Wasted

Although P is abundant in the Earth’s crust (1200 mg kg^–1^) [3], it is dispersed over wide areas. In the soil, its abundance is lower (900 mg kg^–1^ [19]). Moreover, it does not exist as a free element due to its high reactivity, it is therefore frequently bound to chemical compounds making it scarcely available for uptake by phototrophs. The geochemical cycle of P is very different from those of other biogenic elements since there is no cycling through the atmosphere [4]. In contrast to the situation with other macronutrients such as nitrogen and carbon, most ecosystems depend entirely on the aqueous transfer of P [2]. Therefore, the availability of P is the factor limiting the productivity of primary producers such as phytoplankton in most aquatic ecosystems. In turn, microalgae acquired diverse mechanisms to cope with P scarcity during their evolution (Section 2.1).

Likewise, P often becomes the limiting nutrient for terrestrial plant productivity, especially in agroecosystems. Thus, since the time of the Green Revolution, agricultural crop productivity (and hence food security) has depended on intensive fertilizing. Consequently, P fertilizers which are made from rock phosphate are of utmost importance. However, the minable P resources are finite and non-renewable. The estimates of rock phosphate resources are highly controversial, most of them concur on the time span from several decades to several centuries. Moreover, economically extractable deposits of rock phosphate are extremely unevenly distributed between different countries: Morocco controls more than two-thirds of the reserves estimated to be commercially exploitable, while China, Algeria, and Syria possess their shares in the single-digit percentages [20]. Fears of running out of P reserves and thereby jeopardizing global food security continuously resurface, so geopolitics come into play, and the main supply nations could drive up prices, and the consequences of this could be graver than those of competition for fossil hydrocarbons.

More than 90% of the P mined as rock phosphate is currently used in the food supply chain. Paradoxically, the processes in this supply chain are woefully inefficient: more than 80% of the extracted P ends up in waste and is lost in the environment, mainly in the hydrosphere [21]. A part of it becomes bound into poorly soluble chemical compounds, hence it becomes poorly bioavailable, and the remaining can cause its own set of problems such as widespread water quality issues mediated by excessive microalgae growth. Ironically, these microalgae could also provide a means to recover and recycle P as they can store this precious nutrient.

### 1.2. P Acquisition and Storage in the Cell

#### 1.2.1. Cell P Quota and P Uptake Capacity

The capacity of microalgal cells for taking up and accumulating P (cell P quota) is defined by the difference between the lowest and the highest P content [22]. The lowest P content (also called the minimal cell P quota) is typical of microalgal cells whose P reserves are depleted to the largest possible extent. That is, the cell division is slowed down or arrested but the cell sustains no irreversible damage so that its division resumes upon replenishment of P [5,23]. Accordingly, the largest cell P content or largest cell P quota is recorded when all the intracellular P pools (see below) are filled, and no further P uptake is possible in any form. This does not include the P adsorbed on the surface structures of the cell which might be significant; in certain microalgal species such as *Micractinium simplicissimum*, it can even surpass the cell P quota (see, e.g., [24]). Cell P quota can be modulated by the mode of cultivation and the operating parameters [25,26,27,28]. Maximal values of P content of biomass (and per cell) were achieved for *Nannochloropsis salina* in a continuous steady-state mode of cultivation under the highest applied light intensity [29]. In practice, both cell P quota and cell P adsorption capacity are important determinants of the ability of the microalgal culture to capture P from waste streams.

The absolute P content of microalgal biomass under conditions of scarce availability but sufficient for the progression of the cell cycle normally obeys the aforementioned Redfield ratio constituting, on an average, 1–2% of the cell dry weight, e.g., of natural phytoplankton [30]. Interestingly, the proportion of P in biomass of terrestrial plants is much lower due to the large carbon content [21], especially in structural and protective tissues, and, likely, due to the inability to accumulate large quantities of polyPs. The microalgae cultivated under abundant P conditions, e.g., in wastewater, frequently reach 3–4% P in their cell dry weight (CDW) [31,32]. Transient P shortage triggers a complex of mechanisms dramatically increasing microalgal cell capacity of P acquisition, so it exceeds “normal” metabolic demand. This phenomenon called “luxury uptake” (for more detail, see Section 1.2.3) pushes microalgal P content to 7% of CDW and higher [14,15,23,33,34,35]. For example, P-hyperaccumulating microalgae were isolated from the Revolving Algal Biofilm (RAB) system used for P recovery and reported to accumulate up to 14% polyP of CDW for *Craticula molestiformis* [36] which seems to be at the top of realistic P content values. The discrepancy between studies in estimating total P content and, particularly, polyP-P content can be related to the different methodologies used for P metabolites quantification, as other phosphorous molecules (nucleic acids) may interfere with the accurate determination of polyPs [37]. 

#### 1.2.2. Phosphorus Pools in the Cell 

The typical intracellular P*_i_* concentrations in the cell are in the range of 5–10 µM [38,39]. P*_i_* is present almost in all cell compartments where it becomes involved in a plethora of metabolic and regulatory reactions. Although the intracellular P*_i_* concentrations can be estimated as modest, the turnover of intracellular P*_i_* is significant [40]. Intracellular P*_i_* concentration is maintained, independent of external P*_i_* concentration, by the system of P*_i_* homeostasis. This system includes the mechanisms of P*_i_* uptake and the main cell P storage depot in the form of polyPs (discussed in Section 1.2.3 and Section 1.2.4) and several pools of phosphometabolites as further described below. 

**Figure 1:**
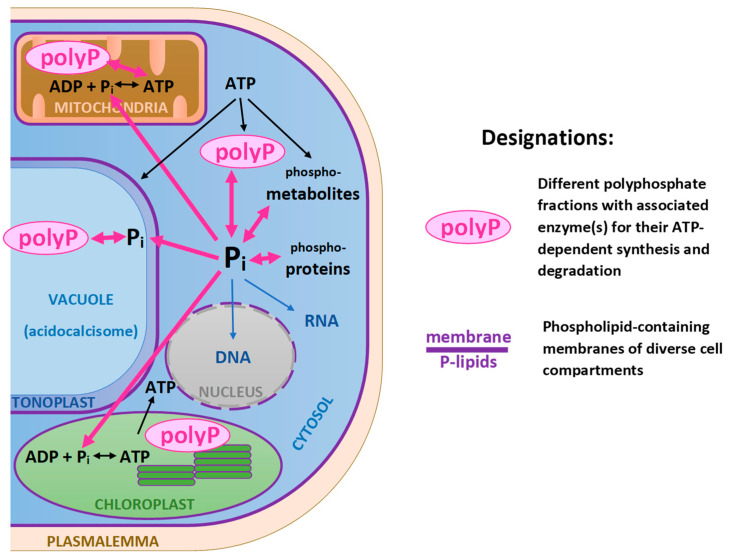
A schematic representation of main P pools in microalgal cells including P*_i_* present in all cell compartments, structural phospholipids of cell membranes, phosphoproteins and phosphometabolites, nucleic acids, and the main storage form of P in the cells, polyPs. For more detail on P uptake, see Section 1.2.3.

In the cell, P exists within different groups of molecules and/or cell compartments designated as P pools (Figure 1). One of the largest P pools is comprised of nucleic acids serving for the storage and transduction of genetic information and developmental signals in living systems. Ribosomal RNA comprises the largest and most flexible P pool whereas the DNA-P pool is quite stable even during P shortage periods. There are also exceptions when chloroplast rRNA increases and chloroplast DNA decreases, during P shortage [41]. This P pool can play an important role in the rapidly growing cultures used for the sequestration of P from waste streams.

A relatively small but functionally important P pool is comprised of phosphoproteins involved in intracellular signaling and regulation, i.e., when the proteins are phosphorylated and dephosphorylated by assorted protein kinases.

Phosphate is a component of a polar group of phospholipids, an important class of membrane lipids. Phospholipids, such as phosphatidylglycerol, occur in plastidial membranes and play an important role in photosynthesis. In higher plants, it is estimated that phosphate is present in less than half of the envelope membrane lipids within chloroplasts, and less than 15% of thylakoid membrane lipids (see [42] and references therein). These estimations can differ in microalgae due to their dynamic nature, complex evolution of membrane systems, and exposure to a rapidly changing environment. Other phospholipids typical for phototrophic eukaryotes can be present in both the plastidial and extraplastidial lipids, as a component of cellular membranes (ER, mitochondria, plasma membrane). Although the content of phospholipids is relatively low compared to other glycerolipids, they are considered an internal P resource important for P sparing under conditions of P scarcity. Additionally, microalgal cells use lipid remodeling strategies to maintain cellular homeostasis under conditions of variable P and environment. Under conditions of P shortage, P-containing lipids can be replaced with non-phosphorous structural lipids, galactolipids, S-containing plastidial sulfoquinovosyldiacylglycerol, or N-containing betaine lipids [43]; implementation of this replacement is genotypically and phenotypically variable [44].

Another large P pool deeply involved in energy storage and transduction is nucleoside triphosphates comprising one of the energy “currencies” of the cell, with ATP as a ubiquitous energy storage form. Overall, a considerable part of intracellular P is also incorporated into diverse P metabolites such as phosphorylated sugars. The main P storage of the microalgal cell is represented by vacuolar polyP which is a dynamic P depot (discussed in Section 1.2.4) whereas in P-sufficient plants, 85 to 95% of P reserves are found as vacuolar P*_i_* [45]. 

#### 1.2.3. Phosphorus Uptake 

While different genes are involved in P acquisition and metabolism within different taxa of phototrophs, the general responses to P shortage are similar to the upregulation of genes responsible for P acquisition, transport, and storage. As mentioned above, P*_i_* homeostasis in the cytoplasm is maintained in the lower mM range by a P*_i_* transport system comprised of intricately regulated mechanisms translocating P*_i_* across the tonoplast. These mechanisms are up- or downregulated in response to changes in the availability of external P and other environmental conditions. Normally, the uptake of P*_i_* takes place against a gradient formed by a high P*_i_* concentration in the cell and a relatively low concentration in the cell surroundings (Table 1), so P*_i_* uptake is carried out mostly by active mechanisms. At the same time, the uptake capacity of this system is limited (for most microalgal species, K*_i_* for P*_i_* uptake is below 4 µM [5]). Loading P*_i_* into the vacuole across the tonoplast also requires ATP [46] and contributes to P*_i_* homeostasis of the cell, especially during luxury P uptake. It is believed that photophosphorylation is the main source of ATP energy for the active P*_i_* uptake in phototrophs, although under severe P*_i_* depletion cyanobacteria can leverage their internal energy sources for P*_i_* acquisition [47]. It was also hypothesized that in marine cyanobacteria, P*_i_* uptake is likely limited by the surface of their outer membrane and not by the cell energy reserves [47].

In marine cyanobacteria, an important role of the periplasm in P*_i_* acquisition has been revealed recently [47]: they acquire P*_i_* by maintaining a periplasmic concentration below environmental levels; the P*_i_* accumulated in the extracellular buffer can then be removed hypo-osmotically by ATP-powered transport. Interestingly, the proton motive force (PMF) is not required for the P*_i_* retention in the periplasm; the loading of P*_i_* into the periplasm across the outer membrane is PMF-dependent and can be augmented by the energy coming from photosynthesis. As a result, marine cyanobacteria can uptake P_i_ even at very low external concentrations, making these organisms good candidates for polishing wastewater with low P*_i_* levels.

**Figure 2:**
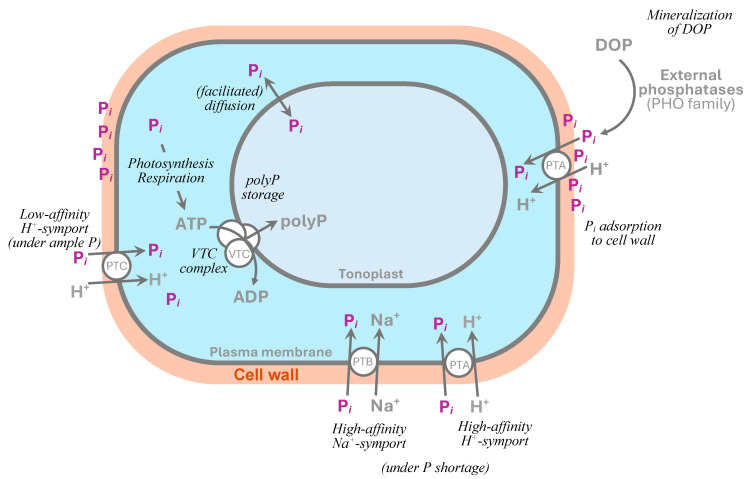
Microalgae including cyanobacteria acquire P with a sophisticated system of Pi transporters operating with a high efficiency across a broad range of external Pi concentrations and environmental conditions. VTC, vacuolar transport chaperone; DOP, dissolved organic phosphorus. For details, see text and Tables S1–S3.

Two main P*_i_* uptake systems of microalgae are represented by two subsystems (Figure 2). One is the high affinity transporters operating when P*_i_* is scarce (normally in natural environments, see Table 1 and Tables S1–S3 for the genes/enzymes involved). These are among the oldest and most highly conserved proteins, more detail on their structure, operation, and evolution can be found elsewhere [45]. Mechanistically, the P*_i_* translocation through the plasma membrane is a co-transport process driven by protons generated by a cytoplasmic membrane H^+^-ATPase. The high-affinity transporters are easily saturated by P*_i_*; in other words, their Michaelis–Menten constant, K_m_ (the concentration of substrate that allows the reaction to proceed at one-half its maximum rate) is low varying from 0.1 to 0.3 μM (as was determined for *C. reinhardtii*). As a result of the deployment of the high-affinity Pi transporters, the V_max_ of P*_i_* transport increases 10–20 times [57]. Important characteristics of the high-affinity P transporters are their rapid and specific induction upon the onset of P*_i_* shortage and repression after re-supplementation of P*_i_* (within ca. 24 h [57]).

Another subsystem is the low-affinity P*_i_* transporters, which function constitutively (i.e., largely independently of the external P*_i_* availability). While the low-affinity P*_i_* transporters cannot acquire P*_i_* when it is present in low concentrations (e.g., PTC1, PTA1), they are not so readily saturated by Pi when it is abundant in the cell surroundings (i.e., they have a higher K_m_ about 10 μM). The low-affinity P*_i_* transporters are therefore responsible for the translocation of the bulk of P*_i_* (ca. 80%) taken up by the cell under abundant P conditions, whereas nearly all P*_i_* uptake takes place via the high-affinity system in P-starved cells [58].

The P*_i_* transporter proteins of terrestrial plants or fungi (yeast) are relatively well known whereas those from microalgae are much less studied; current knowledge of the P*_i_* transporter proteins originates mostly from a handful of model organisms such as *Chlamydomonas reinhardtii* or *Arabidopsis thaliana*. Thus, 25 putative genes—homologs of the higher plant *PHT* family encoding P*_i_* transporters in *C. reinhardtii* divided into four subfamilies of *CrPTA* (H/P*_i_* cotransporter), *CrPTB* (Na/P*_i_* symporter), *CrPHT3*, and *CrPHT4* whose expression is putatively regulated by *CrPSR1* [59]. 

Overall, higher plants and fungi (yeast) harbor genes involved in P*_i_* uptake and its regulation—homologs of the corresponding genes are present in microalgae suggesting the similarity of their regulatory mechanisms.

#### 1.2.4. PolyPhosphate Turnover and Regulation

The maintenance of P*_i_* homeostasis in the cytosolic and other cell compartments is critical for the maintenance of normal cell metabolism which can be easily disturbed by excess of P*_i_*. After a sudden increase in the external P*_i_* concentration (e.g., because of P fertilizer leaching), it may exceed a thermodynamic threshold, so the energy available to the cell will become sufficient for massive P*_i_* uptake. The P*_i_* taken up in excess of the current metabolic demand is stored in the form of polyPs, the relatively metabolically inert and osmotically safe storage form of P [60].

Several organic and inorganic molecules can be classified as polyP(s) with inorganic linear polyP being the main storage molecule. Inorganic polyP is a homopolymer of orthophosphoric acid residues varying in chain length. It has been discovered in all kingdoms of life including oxygenic phototrophs [46,61,62]. In addition to its function as the main P depot of the microalgal cell, it is claimed to serve as a primordial source of energy (stored in phosphoanhydride bonds linking the P*_i_* residues) that may have been used by biological systems prior to the evolutionary advent of ATP, hence it is often referred to as “molecular fossil” [63,64]. Synthesis and accumulation of polyP is related to numerous cellular functions, apart from P storage: it is involved in maintaining adenylate and metal cation homeostasis, counter-ion for cation sequestration, protein activity modulation, and stress acclimation [62,64]. At the same time, uncontrolled synthesis of short-chain polyP can likely mediate P toxicity (Section 2.3). 

Numerous studies on polyP turnover in the cells of microalgae suggest that polyP, as other intracellular reserves, is accumulated (i) when bioavailable P is abundant in the cell surroundings and (ii) when the metabolic demand of P is lower than its influx into the cell. Accordingly, polyP accumulation typically occurs in the microalgal cells early and advances stationary phase when cell division slows down whereas in the cells of rapidly dividing exponential cultures polyP(s) are scarce [15,35]. 

The biosynthesis of polyP is energy intensive; it consumes energy-rich substrates such as ATP or inositol phosphates. While the bulk of energy demand for the biosynthesis of polyP in photoautotrophic cells is satisfied by photosynthesis, the energy for polyP assembly can be partly supplied by respiration or, under anaerobic conditions, by fermentation. Although the latter source is much less efficient in driving polyP biosynthesis [35].

In microalgal cells, polyP reserves are mainly stored in specialized vacuoles called acidocalcisomes [65,66]. According to the current understanding, the bulk of polyP in microalgal cells is synthesized by a complex molecular machinery comprised of several subunits—proteins from the VTC (vacuolar transport chaperone) family [67]. The structure and function of the VTC complex are most studied in baker’s yeast (*Saccharomyces cereviseae*) where it is a sophisticated protein complex assembled from the polyP polymerase VTC4 and location-specific combinations of the accessory VTC1, VTC2, VTC3, and VTC5 subunits [68,69]. In yeast, VTC was found to be activated by the binding of inositol pyrophosphate with the participation of (presumably) the P*_i_*-sensing SPX domain [67], a domain discovered in many other genes involved in P*_i_* metabolism. Genomes of many microalgal species harbor genes encoding VTC complex subunits and recent in silico evidence showed that VTC4 proteins are structurally highly conserved [70] suggesting that the VTC complex is broadly distributed in microalgae and commonly involved in polyP synthesis in their cells [14,15]. The structure and mechanism of VTC operation in microalgae remain underexplored [32,70,71].

In cyanobacteria, polyP is synthesized by polyPhosphate kinase 1 (PPK1 which is evolutionary unrelated to VTC of eukaryotes [64]), degraded by polyPhosphate kinase 2 (PPK2), an enzyme with reversible activity, and the exopolyPhosphatase (PPX) cleaving the polyP units. While homologs of PPK2 and cytosolic PPX, as well as other enzymes with documented polyP hydrolase activity, were not found in the proteome of *C. reinhardtii* [70,72], a diadenosine and diphosphoinositol polyPhosphate phosphohydrolase (DIPP) enzyme was suggested to be involved in polyP degradation in *C. reinhardtii* [72]. Notably, variation of *ppk*/*ppx* gene expression might be a very flexible mechanism of acclimation of cyanobacteria to diurnal variation of P*_i_* availability [73], so this mechanism can be of importance for biosequestration of Pi, e.g., from waste streams with varying P*_i_* abundance. 

Regulation of polyP biosynthesis in microalgae experiencing sudden fluctuations of P*_i_* availability appears to be very dynamic. Thus, the cells of *Synechocystis* can be “primed” for luxury uptake of P*_i_* and storage even by a short period of P deficiency (polyP accumulation took place in the first 1–3 min after the replenishment of P*_i_*). In eukaryotic microalgae, these processes generally take more time but follow the same pattern (see, e.g., [15]). The accumulation of polyP can be triggered by other limitations (e.g., by sulfur deficiency [74]) slowing down the cell division rate and the corresponding P*_i_* expenditure. The polyP content of cyanobacterial biomass can also be enhanced by slowing down its degradation under abundant P conditions by knocking out the *phoU* gene, a negative regulator of P*_i_*-responsive genes in bacteria sensitive to the external P*_i_* level [75]. This approach can be beneficial from the standpoint of technology since it would allow retention of the high polyP content in the grown biomass, which normally tends to decline upon resumption of cell division after the transient polyP accumulation following P*_i_* refeeding of the culture.

Importantly, in eukaryotic algae, a shortage of bioavailable P readily upregulates the synthesis of the components of the VTC complex rendering the cell capable of rapid polyP accumulation whenever P*_i_* becomes available. Proteomics studies revealed that the turnover time of the proteins involved in polyP biosynthesis is on the scale of hours since in *C. reinhardtii*, polyP biosynthesis continues when the genes encoding VTC subunits 1 and 4 (*Cre12.g510250* and *Cre09.g402775*) are already downregulated [71], suggesting the involvement of post-translational regulation in the control of polyP biosynthesis by the VTC complex, at least in *C. reinhardtii*. The similarity of the response of many species from the transition to P depletion to P repletion suggests similar regulation in eukaryotic microalgae [71].

It should be also noted that many proteins involved in polyP turnover harbor SPX domains thought to be responsible for sensing P*_i_* levels in the cell. Thus, polyP synthesis is thought to be stimulated by binding inositol pyrophosphate to the SPX domain of the VTC4 subunit as was revealed in VTC4 of *C. reinhardtii* [64,71,72] as well as *Chlorella vulgaris*, *Desmodesmus armatus,* and *Gonium pectorale* [70]. 

The evidence on the effects of polyP on resource allocation and the ability of the culture to accumulate biomass are controversial. On one hand, there is a consensus that polyP biosynthesis is energy-intensive. Indeed, the knockout of the *ppk* gene encoding polyP-kinase increased the biomass productivity of *Synechocystis* sp. under favorable conditions [76]. On the other hand, in certain eukaryotic microalgae (e.g., *C. reinhardtii*, *C. vulgaris*, and *D*. *armatus*), growth was not negatively affected by P assimilation and polyP synthesis [70].

Overall, cells and cell populations of cyanobacteria and microalgae are heterogeneous in their ability to accumulate and metabolize polyP to adapt to fluctuating P*_i_* availability [77]. They may adopt different strategies encompassing either slow growth with a high amount of stored polyP or fast growth and cell division at the expense of the stored polyP reserve [77].

## 2. Between Scylla and Charybdis: P Starvation and P Toxicity

In nature, microalgae experience mostly oligotrophic conditions. It means that most of the time microalgae are facing scarce and fluctuating availability of key mineral nutrients including phosphorus. This is the case for the habitats with very low P availability such as the oligotrophic central oceanic gyres [78], and very old soils found, e.g., in Western Australia and South Africa [2]. As a result, microalgae are naturally equipped to cope with P scarcity by multiple mechanisms they developed during their evolution. Evolutionary adaptation to P shortages has left a deep mark on the genomic landscape of oxygenic phototrophs manifested, e.g., by diverse regulation mechanisms affecting a wide range of genes triggered by a decline in P availability [13,79]. Briefly, these mechanisms enable the microalgae to obtain as much of the bioavailable P as possible and to do it as quickly as possible. The intracellular P is then neutralized during storage and can safely be stored for future P shortage.

By contrast, the mechanisms allowing the capacity to cope with extremely high nutrient spikes that may be harmful (see Section 2.3) are apparently lacking in microalgae [14,23]. Abrupt increase in nutrient availability either due to natural reasons (upwelling, runoff after storms, etc.), or anthropogenic (wastewater discharge, fertilizer run-off from the fields, etc.), reasons lead to harmful algal blooms (HABs), toxicity issues, and eutrophication. 

Situations where fluctuating availability of P occurs can be problematic in microalgal biotechnology. Indeed, the mineral nutrient composition of many waste stream types is not balanced in terms of N:P ratio leading to incomplete removal of either nitrogen or P (Section 3.2.2), other challenges may arise during the treatment of wastewater with very high content of P, e.g., those from phosphate mines and/or rock phosphate processing plants.

### 2.1. Phosphorus Starvation

The phenomenon of P starvation in microalgae is relatively well studied. This is because a significant research effort was directed at revealing the dynamics of phytoplankton in the aquatic ecosystems which is believed to be modulated, to a large extent, by limited P availability [13,45]. Most of the studied microalgal species are capable of accumulating abundant reserves of P (Section 1.2) sufficient to support the cell division for several generations (up to 20 [80]) in the absence of external bioavailable P. Moreover, as further described below, the cellular plasticity to P shortage allows the cell to survive for many days. Therefore, deprivation of P is considered to be a relatively mild stress in comparison with, e.g., nitrogen deprivation [81]. Deprivation of P leads to activation of several acclimatory mechanisms which can be divided into two types: (i) the mechanisms increasing the efficiency of P acquisition by the cell and (ii) the mechanisms of mobilization of intracellular P reserves and P sparing mechanisms such as membrane lipid remodeling [43,71]. 

The first is the changeover of the P*_i_* transporters in the cell membrane: the high-affinity P*_i_* transporters genes which (Section 1.2.3) are upregulated, lead to an increase in high-affinity P*_i_* transporters, replacing the low-affinity transporters. As a result, the cell increases its capacity to fetch P*_i_* at its low external concentrations, though at a cost of additional expenditure of energy. Then, the expression and secretion of the extracellular enzymes such as purple phosphatase(s) capable of liberation of P*_i_* from DOP is increased (Section 2.2) so the capability of the cell to acquire P from its surroundings is enhanced.

The second is salvaging P from cell components such as expendable types of nucleic acids including ribosomal RNA and other surplus RNA molecules [71]. The total rRNA declines during P shortage; it can drop to the level of the P content within DNA as was demonstrated for the marine picoplanktonic cyanobacterium *Prochlorococcus marina* [82]. The pools of P*_i_* in different cell compartments as well as P metabolites such as sugar phosphates are also being gradually depleted [83,84]. 

Another critical mechanism enabling microalgal cell metabolic plasticity in response to limiting P is membrane lipid remodeling [85]. In higher plants, the well-studied response to P limitation constitutes the replacement of phospholipids with non-phosphorous galactolipids, namely the extraplastidial phospholipids with a bilayer forming galactolipid digalactosyldiacylglycerol (DGDG), and in the plastid, the replacement of the acidic phosphatidylglycerol with sulfoquinovosyldiacylglycerol (SQDG) to sustain photosynthetic activity [86,87]. This strategy is also used by microalgae and an increased ratio of SQDG to PG was reported as a biomarker of P-limited oceanic waters [88]. The replacement of degrading phospholipids with certain galactolipids, betaine lipids, and sulfolipids was also shown in the microalga *C. reinhardtii* and *Nannochlorpsis oceanica* [43,71].

In P-replete cells, the predominant lipid classes are non-phosphorous plastidial glycoglycerolipids, which include monogalactosyldiacylglycerol (MGDG), DGDG, and SQDG. Phosphorous-containing membrane lipids include phosphatidylglycerol (mainly present in the plastids) and several classes of phospholipids, involved in the key reactions of membrane and storage lipid biosynthesis [15,43,89,90]. Importantly, many eukaryotic microalgae contain an additional class of membrane lipids—the non-phosphorous betaine lipids [91]. This group of membrane lipids was deemed to be lost in higher plants during evolution along with the establishment of a sessile lifestyle [92]. Betaine lipids are present in many groups of microalgae and are represented by three main types. Diacylglyceryl-N,N,N-trimethylhomoserine (DGTS), abundant in green microalgae, has structural similarity to phospholipid phosphatidylcholine, PC. Its content generally increases along with galactolipid DGDG under conditions of P scarcity, while the content of phospholipids decreases. Recent biophysical studies suggested that bilayers formed by DGTS have some beneficial features in terms of thickness and water repulsion [92]. The capacity to swiftly remodel membrane lipids in response to P availably may have important consequences for P luxury uptake and P retaining in the cells. This direction warrants further studies. To note, P starvation responses are regulated in the cell at different levels, including the transcriptome level, and regulation of many transcriptional factors and lipid metabolism genes. Overall, survival and growth of microalgae under P deprivation or limitation needed for obtaining low cellular P quota, are tightly related to lipid metabolism. 

Lipid remodeling has also been shown to be a key component of cellular autophagy during P shortage [89]. Autophagic degradation of cellular components supports cell homeostasis during nutrient starvation [93,94]. Autophagosome formation relies on membrane modeling and re-modeling events, from the nucleation of the phagophore to its expansion and closure [95]. Autophagy may play a role in response to P deprivation, which is associated with a decrease in energy-rich molecules such as ATP, and a range of phosphorylated metabolites [89,96]. Phospholipids are required for the construction of autophagosomes and the operation of autophagy flux at several levels [97,98], e.g., for lipidation of the key autophagic protein ATG8 (a ubiquitin-like protein [99]). The lipids associated with the autophagosome have started to be revealed in plants. While a new player in plant autophagosome formation appeared to be a negatively charged phosphatidylinositol-4-phosphate (PtdIns4P) [95], the lipid(s) involved in microalgal ATG8 lipidation is currently unknown. Because autophageous vacuoles decline with the duration of P starvation, we hypothesize that prolonged P deficiency depletes phospholipids required for the autophagy machinery and thus cells are unable to completely degrade polyP reserves. 

Critically, the responses to P shortage are complex and cells have been shown to “prioritize” strategies for P homeostasis. For instance, *P. shikokuense* prioritizes the preservation of RNA and polyP for crucial metabolic processes while sparing and recycling phospholipids with non-phospholipids. Concurrently, autophagy is triggered in *P. shikokuense* under phosphorus deficiency, thereby decreasing energy exhaustion and potentially conserving and releasing phosphorus resources for more vital metabolic pathways.

However, the microalgal cells can retain some polyP reserves even after long P starvation (30+ days), the reasons for this remain enigmatic [84]. A plausible hypothesis explaining the loss of the ability of the cell to mobilize its polyP reserves is the failure of autophagic mechanisms. The latter can result, e.g., from insufficient energy supply due to an overall slowdown of metabolism under the P shortage stress. On the other hand, polyPs are molecules with many metabolic functions (as mentioned earlier in the review), so it is possible that not all polyP is consumed. This evidence that the regulation/sensing of P levels (as well as the difference between species) needs to be elucidated.

Eventually, when all P resources, both internal and external, are exhausted, the cell division stops, and the typical rearrangements of nutrient-starvation stress in microalgae take place. Briefly, these are the reduction of photosynthetic apparatus (decline in photosynthetic pigments such as chlorophylls, phycobilins, and primary carotenoids and dismantling of the thylakoid membranes) to avoid excessive light capture and hence the photooxidative damage when light and dark reactions of photosynthesis are off-balance [100]. Another hallmark of changes in the cell induced by nutrient starvation including P starvation is the accumulation of carbon reserves, mostly in the form of starch and neutral lipids. Accordingly, nutrient limitation/deprivation is frequently used to control the biochemical composition of industrially cultivated microalgae. However, as it was noted above, the P starvation stress is milder than, e.g., nitrogen starvation [101] and hence is less manageable.

As a net result of acclimation to P shortage, microalgal cells gain the capability for rapid uptake of P*_i_* in large qualities. On one hand, this capability is thought to be advantageous: the species that captures P from the environment faster than others can thereby starve its competitors [102]. On the other hand, there is a danger of P*_i_* intoxication mediated by short-chain polyPhosphates (see Section 2.3).

### 2.2. Mobilization of External DOP

Like higher plants and heterotrophic microorganisms, most microalgae [84,103,104,105] possess the ability to express external phosphatases under P shortage conditions facilitating the mobilization of P from the dissolved organic molecules in the cell surroundings (commonly termed dissolved organic phosphorus, DOP). In the model microalga *C. reinhardtii*, several extracellular and cell-wall-associated phosphatases were discovered, including those constitutionally expressed and the alkaline and neutral phosphatases induced by limited P conditions, PHOX [79].

Omics studies revealed diverse phosphatases, many of which may be responsive to environmental P levels in many other microalgal species such as coccolithophore *Emiliania huxleyi*; dinoflagellates *Karenia brevis*, *Amphidinium carterae*, and *Alexandrium catenella* (reviewed in [13]). At the same time, reservations should be made regarding the relevance of these findings since it is relatively easy to identify a putative phosphatase gene, and even show that transcripts from the gene respond to the environmental P status, but it is more difficult to reveal the cellular location, kinetic features and, ultimately, the actual function of a putative phosphatase in the P metabolism [106]. 

Clearly, the ability to mobilize external DOP is very important for the treatment of organic-rich waste streams like those from diverse food industries. So, the strains with a large repertoire of efficient external phosphatase should be prospected for this application. The ability of microalgae to grow and store P from organic sources during biotechnological processes has not been investigated. Considering that they possess the machinery for it, this should be investigated in the future as it was conducted in yeast [107]. 

### 2.3. Phosphate Toxicity and Resilience to Elevated P_i_ Concentrations

A literature survey indicates that the studies of microalgae under conditions where P is in large excess of the cell demand are much scarcer than studies focusing on P starvation. Accordingly, the phenomenon of P*_i_* toxicity remains largely underexplored. Therefore, understanding the mechanisms related to P repletion is important for the development of microalgal treatment of waste streams with high P load, e.g., those from phosphate mining sites or fertilizer production plants. It is important to realize that the deteriorative effects of excess P*_i_* on microalgae are beyond mere osmotic stress. Recently, reports described the inhibitory or toxic effects of high external P*_i_*, e.g., in *Chlorella vulgaris* [108]. In these reports, the P*_i_* toxicity was putatively associated with the formation of abundant fine-grained polyP inclusions in the microalgal cells incubated at high external P*_i_*.

Current reports on P metabolism in microalgae do not suggest the existence of specific pathways for the detoxication of P*_i_* when it presents in high concentrations. Extracellular structures like cell walls with high P*_i_* adsorption capacity can contribute to elevated P*_i_* resilience. Another determinant of elevated P*_i_* resilience is the ability to throttle its influx into the cell by downregulation or gating of P*_i_* transporters. 

To prevent the disturbance of the metabolism by P*_i_* which has been taken up already, it should be converted into a “safe”, less osmotically and metabolically active form of polyP and isolated in a storage cell compartment such as the vacuole. However, biosynthesis of polyP requires a large investment of energy, mainly in the form of ATP which may not be readily available, especially under stressful conditions limiting the metabolic resources of the cell.

In view of the phenotypical hallmarks of high P*_i_* toxicity, it was hypothesized that at high P*_i_* influx, synthesis of many polyP chains can be initiated simultaneously, but due to limited availability of energy, it never completes resulting likely in the formation of abundant short-chain polyP. As short-chain polyP can interfere with protein folding and possibly other processes in the cell, their accumulation can result in a slowdown of the cell division and other signs of inhibition observed under large excess of P*_i_* [109,110]. 

This hypothesis was corroborated by the appearance of diffuse signal attributable to polyP on the EDX spectra taken from the cytoplasm of the cells exposed to a high P*_i_* level in the absence of visible polyP formation [108]. Moreover, the failure of high P*_i_* tolerance in microalgae was observed upon abrupt P*_i_* re-feeding of the microalgal cultures pre-starved of P and hence expected to be metabolically quiescent; notably, this was the case even upon the addition of P*_i_* in concentrations well below the toxic level [108]. An abrupt transition from P shortage to abundant P conditions leads to a dramatic increase in P*_i_* influx into the cell (since 10-fold increase in the V_max_ of the Pi transport to the cells). Interestingly, the symptoms of P toxicity also developed in higher plants upon an abrupt transition from P-depleted to P-replete conditions [111]. This needs to be taken into consideration for the development of bio-processes for P upcycling.

## 3. Biotechnological Implications

### 3.1. Microalgae: The Curse of Eutrophication and the Boon of Biosequestration

As noted above, in nature, P*_i_* slowly enters aquatic ecosystems, remains in the water column for a long time (from a few to thousands of years, and ends up sedimented on the ocean and lake bottoms (see [2,78] and references therein). There, it is recycled at an extremely slow rate subject to the action of many environmental factors including pH, redox potential, and salinity [78]. Anthropogenic intervention dramatically changed the distribution of bioavailable P*_i_* in the environment. Thus, according to recent estimations, human activity mobilizes as much phosphate each year from rock phosphate deposits and other sources as is mobilized by “natural” processes from base rocks in soil genesis [112]. One of the reasons for this is the massive application of P fertilizers. Much of the P*_i_* applied with the fertilizers to the soil becomes unavailable to plants because it either becomes immobilized (changes chemically) even though it stays in the soil or is washed away from the root zone [2]. Unfortunately, there are no known technologies for preventing such diffuse P losses in the environment. Although microalgae cause detrimental HABs under eutrophic conditions, they are increasingly claimed as an efficient vehicle for recovering P from point sources such as sewage and waste from animal farming. Microalgae are believed to be capable of closing the P loop by converting the P-rich microalgal biomass into biofertilizers, supplements to animal feed, and other valuable bioproducts. The suitability of microalgae for this role stems from their adaptations to fluctuating P availability in the environment acquired during their long evolution under such conditions (see below).

### 3.2. Microalgae-Mediated Biocapture of P

The findings reviewed above suggest that microalgae per se are very capable organisms regarding the uptake of different forms of P and using microalgal biomass for biocapture of P from waste streams is arguably promising. Although there are established practices of P bioremoval from waste streams with heterotrophic bacteria such as enhanced biological phosphorus removal (EBPR) to manage eutrophication risks [113], these are still technologically complex and expensive, hence unaffordable by small communities and companies. At the same time, the use of microalgae for biocapture of P is advantageous because of (i) more efficient wastewater treatment due to photogenerated oxygen [114]; (ii) generation of biomass convertible to valuable products such as biofertilizer (Section 3.3); (iii) simplicity with low capital and operational costs (low energy requirements), consequently having a lower environmental footprint than other alternatives [17].

Still, practical implementation of this approach can be problematic without a deep understanding of the relationships between culture operational parameters (cell density, growth phase), cultivation conditions (light, carbon and other nutrient supply, temperature, pH, mixing, presence of toxicants, etc.), and the kinetics of P uptake translating into the amount of P that can be sustainably removed from the medium per unit of time. Of special importance, e.g., for wastewater polishing, is the completeness of P removal. Finally, the opportunities for the utilization of P-rich biomass obtained during P biosequestration are of crucial importance.

From the standpoint of technology, it is important to realize how large the potential of microalgal culture for P removal under specific conditions and constraints. There are many reports in the literature presenting specific figures on P uptake under diverse experimental setups and a broad range of wastewater compositions (for a recent summary, see Table 1 in [17]), however, it is more difficult to find a summary relating the culture conditions and the efficiency of P bioremoval, above all under full-scale conditions. In view of this, we attempted to generalize the reported data to infer general prerequisites for efficient bioremoval of P from waste streams using microalgae.

#### 3.2.1. Cultivation Conditions and P Nutrition History of the Culture

The natural acclimations of microalgae to fluctuations of P*_i_* in the environment including the dramatic increase in P uptake capacity and overplus response (Section 2) can be exploited to achieve the highest P*_i_* uptake rates and P contents of the resulting biomass [32,70]. Moreover, nearly complete removal of P from the medium can be achieved induced by P pre-starving of the culture [28]. 

Knowledge of the kinetics of P*_i_* uptake and conversion into polyP is important to obtain microalgal biomass enriched with polyp, which represents a valuable biofertilizer. Thus, the cyanobacterium *Synechocystis* sp. PCC6803 accumulated polyP after 3 min of exposure to abundant P*_i_* conditions whereas 1 h later polyP content started to decline [77]. Similarly, the transient increase in polyP in P-starving *Chlorella vulgaris* IPPAS C-1 cells was observed 4 h after a P*_i_* spike [15]. This capability of luxury P*_i_* uptake renders microalgae particularly suitable for dealing with the spikes of P*_i_* concentration in waste streams.

Importantly, the polyP accumulation depends on the energy coming (in the form of ATP) from photosynthesis and it is sensitive to extreme temperatures [35,77]. Therefore, the adequate supply of light energy to the phototrophic cultures is important to ensure efficient polyP accumulation. However, these requirements may be difficult to fulfill as the wastewater is turbid. Since atmospheric CO_2_ levels are limiting for microalgal growth, enrichment of microalgal cultures with CO_2_ augments their growth thereby boosting their P uptake capacity [115] although this requirement may be less strict in organic-rich wastewater and/or microalgae capable of mixotrophic growth. A decline of pH by injection of CO_2_ also increases the P_i_ bioavailability for microalgae [116,117], although care should be taken to keep the pH within the range suitable for the microalgal culture.

The P-sufficient vigorously dividing cultures continue to consume P at a steady rate, and the sustained bioremoval of P*_i_* is currently the mainstream approach in environmental applications [17,118,119]. Although the absolute amount of P removed by such cultures can be significant, complete P removal from the medium of dense P-sufficient cultures is difficult to achieve because of the release of P into the medium, e.g., from dead cells. This is a common reason for the notoriously low efficiency of dense microalgal cultures at the removal of low amounts of P (typical completeness of P removal is around 90% at an initial P load of 4–30 mg L^–1^ [17]). 

To achieve both goals of polyP-rich biomass production and removal of P from treated wastewater, a biphasic approach has been suggested when the bulk of P is removed by P-sufficient microalgal culture and the polishing of the effluent is carried out by a slightly P-starved culture [14,28]. Based on the fundamental metabolic knowledge discussed in Section 1.2.3 and Section 1.2.4, such systems would indeed trigger polyP synthesis and P uptake via the upregulation of P transporters and VTC proteins. 

#### 3.2.2. Algal–Bacterial Communities and P Acquisition

Microalgae and bacteria are already forming a successful synergy in waste stabilization ponds (WSPs), with microalgae providing the oxygen needed for bacteria to degrade organic compounds. To the best of our knowledge, the extent of this synergy in relation to P is unknown. Bacteria can play a key role in P availability in soil, rendering P bioavailable through pH changes, however, P bioavailability should not be an issue during wastewater treatment. The environmental conditions in algae-based wastewater treatment systems are such that most of the P storage would be performed by the algae. However, many factors including the availability of dissolved organic carbon can affect the efficiency of microalgae and cyanobacteria by modulating their relationships with heterotrophic bacteria and recycling of P released after cell death as was recently established for Microcystis [120]. A promising approach to using the microalgal–bacterial co-cultures taking P from wastewater as biofertilizers is comprised of their immobilization on biodegradable carriers [121].

#### 3.2.3. Phosphorus Load and Nutrient Balancing

Generally, the capability of microalgal culture to take P is determined by its cell P quota and cell division rate which can be limited by stresses like other nutrient shortages, cell shading, or extreme temperatures. This translates into the requirements of optimal nutrient load and composition within the wastewater treated with the microalgal culture. Bioprocess for P biocapture with microalgae should be developed taking into account the available cell P quota and the amount of P to be removed: as mentioned above, over-saturated cells with their P quota full will not take up P*_i_* even if it is available, and the conditions are favorable. Furthermore, the rate of the nutrient loading on the culture should match its growth rate and cell P quota size, otherwise, the efficiency of P bioremoval will decline. Thus, nearly complete P*_i_* removal can be easily achieved at a P load around 5–10 mg L^−1^ but increasing P load above ca. 70–100 mg L^−1^ results in the decline of the removal efficiency to 80–90% [17] (however, there can be more than one reason for such a behavior, see below).

Unfortunately, most of the reports on P removal of microalgae seldom include the determination of cell P quota size (i.e., “benchmarking” of the cultures regarding their P*_i_* uptake capacity). Moreover, current summaries of these reports lack indication of actual culture density (cell number and/or biomass content) and pre-cultivation conditions (see, e.g., [122,123,124]). Consequently, it is difficult to infer the actual efficiency of the microalgal cultures at P bioremoval and it is even more difficult to compare results obtained in different studies with the same algal species and wastewater types. 

Waste streams are frequently characterized by an imbalance of the key nutrient (P, nitrogen, organic, and inorganic carbon) composition differing considerably from their proportions of the Redfield ratio [16]. Particularly, nitrogen (more often) or P can be in excess, so to achieve a complete bioremoval of the nutrients the limiting one should be supplemented. An alternative approach comprises the selection of microalgal species/strains with a smaller N/P ratio in the biomass matching that of the wastewater more closely [119]. The P uptake capacity of microalgal cultures experiencing a shortage of nitrogen, sulfur, or other essential elements shortage will be low as well. Still, the relatively low amounts of P*_i_* which are taken up under conditions limiting cell division rate can be converted into polyP more efficiently [66,74]. In this situation, the accumulation of polyP per cell might increase [46] but the overall culture productivity will decline. On the other hand, P shortage does not seem to severely limit the bioremoval of nitrogen [125].

Unforeseen problems may arise during the treatment of wastewater contaminated by hazardous micropollutants and/or heavy metals. Although abundant Pi can be, in principle, beneficial for the contaminant resilience of algal–bacterial communities in treatment facilities, toxic effects of the pollutants can deteriorate their P recovery capacity [126].

#### 3.2.4. PolyP and Stress Resilience in the Context of Waste Stream Bioremediation with Microalgae

As noted above, polyP due to its polyanionic nature functions as a counter-ion for metal cation sequestration, mainly in the vacuole. Normally, this function is carried out for physiologically relevant cations such as Na^+^, K^+^, Ca^2+^, or Mg^2+^ [66]. At the same time, polyPs were shown to participate in the detoxication of heavy metal cations, both required for essential cell functions (Mn^2+^, Zn^2+^, Fe^3+^, Cu^2+^) and toxic metals uninvolved in the cell metabolism (Cd^2+^, Hg^2+^, Pb^2+^) and even radionuclides such as cesium and uranium [127,128,129]. Overall, the reports summarized in [64] are consistent with the existence of the relationship between polyP accumulation and tolerance to high external concentrations of heavy metals based likely on the ability of polyP to bind metal ions and thereby detoxify them. This relationship can be important for bioremediation of waste streams containing, apart from P, high amounts of heavy metals as is the case for rock phosphate mine leachate.

PolyP has also been suggested to participate in the mitigation of excessively alkaline pH harmful for photosynthesis and ATP generation which might arise in microalgal cells incubated in the presence of a high concentration of ammonium [130]. This capacity is especially relevant for bioremediation of specific wastewater types like dairy wastewater and aquaculture without biofilters rich in ammonia. There are also indications of polyP participation in the resilience to osmotic stress in microalgae by the maintenance of ATP levels [131,132].

### 3.3. Microalgal Biomass Is an Efficient and Environmentally Friendly Biofertilizer

The waste streams rich in macronutrients including P and organics cannot be applied for irrigation or fertilization directly because of the presence of hazardous micropollutants (e.g., antibiotics) and other substances endangering the soil as well as public health. This is reflected by the current legislative ban on application of the untreated wastewater in the fields. On the other hand, a novel German regulation on P recovery from wastewater treatment plants prescribes that the recycling of 50% of P from sludge and 80% of P from ash should be achieved by the year 2029 [6]. 

Over the past decade, a consensus built up regarding the feasibility of closing the P loop by upcycling waste streams with microalgal cultures [7]. One way to achieve this is to restrict the use of conventional P fertilizers by increasingly substituting them with biofertilizers from P-rich microalgal biomass. This approach looks attractive above all when alternatives such as increasing P use efficiency either by purely conventional and/or genome modification-assisted breeding are challenging.

The effects of biofertilizers from microalgal biomass have been systematically studied for around two decades, although the studies dedicated to P are markedly scarcer than those focused, e.g., on nitrogen fertilization (see [133,134,135] and references therein). It became clear in general that this type of biofertilizer has distinct advantages over traditional chemical fertilizers [18]. First of all, it is the rate of the release P available for the uptake by plants which, in the case of microalgal biomass, turned out to be close to the rate of P uptake by crop plants [136]. This makes the microalgal biomass a natural analog of the expensive man-made controlled-release fertilizers. Another important advantage is on the side of agrotechnology: microalgal biomass can be applied on the soil surface without tillering into the soil resulting in considerable savings of fuel, and labor, and reduced impact on the soil ecosystem.

As fertilizers are intended to support soil fertility and increase the crop harvest size (see, e.g., Table 2 in [18]), it is important to ensure that microalgal biomass satisfies these requirements. Indeed, recent experiments with nutrient-poor soil substrates showed that (i) the P from the microalgal biomass is nearly quantitatively transferred to the soil and then recovered by crop plants increasing available P in the soil 2–5 times [18] and (ii) the growth of the plants fertilized with microalgae was commensurate to that of the plants fertilized by conventional P fertilizers [136]. Fertilization with microalgal biomass is also expected to replenish the soil pool of bioavailable microelements such as Zn, Mn, and Cu, which is depleted by intensive plant cultivation thereby preventing further limitations of these elements. Despite a considerable number of optimistic reports, the capability of the microalgal biomass as a substitute for the conventional P fertilizer depends on many factors such as crop type, soil, agricultural practices, climatic conditions, etc., so it must be evaluated on a case-by-case basis [137].

Additional beneficial effects on crops frequently arise due to hormone-like substances synthesized and released by the microalgae and, likely, their bacterial symbionts [135]. Strictly speaking, these effects cannot be termed biofertilizers (since they are not related to the bulk inflow of macronutrients and bioactive molecules to the soil). Application of microalgal biomass also increases the bioavailability of the P initially present in the soil [134] and improves its cycling [135]; however, this effect is of secondary importance in the context of this review, further details can be found elsewhere [135]. 

As reviewed in [135], the exogenous polysaccharides (EPS) produced by cyanobacteria and specific microalgae improve soil physical properties in agricultural settings, stabilize soil, and form additional pores. The effect related to water holding capacity was especially evident in experiments with inoculating low-organic carbon soils. In view of these findings, microalgal biomass applied as P biofertilizer is expected to exert beneficial effect(s) on the soil and crop condition. Indeed, no adverse changes either in the soil microbiome or in its activity were found following the fertilization with microalgal biomass [138].

Finally, fertilizers should not interfere with soil microbial activity and other parameters commonly designated as “soil health”. Microalgal biomass application was found to be beneficial for soil health by increasing the organic carbon content of the soil without measurable enhancement of greenhouse emissions except for CO_2_ [134,139]. Still, the data on the greenhouse gas emission from the soil fertilized by microalgal biomass remain controversial [18,140] as well as the comparative estimations of the integral environmental impact by the conventional (e.g., triple superphosphate containing ca. 45% of P_2_O_5_ equivalents in the form of Ca(H_2_PO_4_)_2_) and microalgal biofertilizer production [141].

Although the evidence supporting the suitability of microalgal biomass for the production of green P-rich biofertilizers, biostimulants, biocontrol agents, and soil conditioners is growing, there is a need for further research in this direction. Thus, the rate of microalgal biomass supplementation to conventional P fertilizers such as triple superphosphate to reduce the application rate of the latter requires precise optimization to avoid adverse effects of biomass on the dissolvability of the superphosphate [118,142]. 

Another concern is the requirement of CO_2_ enrichment of the cultures to achieve a realistic growth rate, but the demand for the inorganic carbon can be satisfied by nearby point CO_2_ sources such as flue gases from, e.g., thermal powerplants [143,144,145]. Although leveraging CO_2_ from coal-burning power plants will require additional treatment of the flue gases to remove the excess sulfur oxides and traces of heavy metals, the facilities powered by natural gas burning can be a cleaner source of CO_2_ suitable for direct injection to the culture [146]. Of special interest in the context of supplementation of the microalgal cultures with inorganic carbon are biogas-generation facilities [147]. Thus, using microalgae for biogas upgrading will simultaneously remove the excess CO_2_ from the biogas and feed the culture with inorganic carbon without the risk of contamination of the algal biomass with heavy metals [148,149,150].

Critically, the technologies for the economically viable large-scale growing of P-rich microalgal biomass, its preservation, and transportation are not yet established [32]. Another possible limitation stems from the economic viability and high energy consumption of microalgal-based biofertilizers. It is estimated as 6.51 kWh per 1 g of recycled P, so the use of photovoltaic solar energy during bioprocessing [18,140] will increase competitiveness with conventional fertilizers in the near future. One way to improve the cost-efficiency of biofertilizers from microalgal biomass is the co-cultivation of microalgae with nitrogen-fixing bacteria to cut down the expenses for nitrogen fertilizers [151,152]. However, the ability of the diazotrophic cultures to accumulate biomass might be limited since nitrogen fixation is a very energy-intensive process. Therefore, finding the appropriate balance between nitrogen fixation and overall culture productivity requires further research.

The production of biofertilizer from wastewater-grown microalgal biomass requires strict safety control to exclude the contamination of the biomass and, subsequently, soil by hazardous micropollutants. The latter include heavy metal ions, microplastics, drugs, and nanoparticles which became increasingly widespread in the waste streams all over the world and are readily taken up by microalgae [153,154,155].

Admittedly, the development and application of microalgae as P biofertilizer needs further research which will be fueled by the expansion of their global biofertilizer market forecasted for the coming years (to USD 3.1 billion by the end of 2024 [156]). 

### 3.4. PolyP as a Valuable Commodity

PolyP, due to its unique properties and relatively low cost of synthesis, nontoxicity, and biodegradability, is widely used in various industries (see above and [6,157]).

In addition to the functions of polyP within the cell (Section 1.2.4), polyP also appears to be involved in symbiotic and parasitic associations. In higher non-photosynthetic eukaryotes, polyP levels were proposed to moderate host-pathogen interaction [158], play a role in cancer cell proliferation, and apoptosis, exert procoagulant and proinflammatory effects, and disrupt TOR signaling (see [64,159] and references therein). PolyP also participates in bone tissue development, they are promising candidates in therapy for bone and blood diseases [159]. Accordingly, polyPs are considered for application in developing novel bone substitute materials, carriers for prolonged action pharmaceuticals, and donors of P for enzymatic syntheses of biologically active compounds [160]. 

PolyPs are widely used as reagents in water treatment, fertilizers, flame retardants, and food additives due to their unique properties (reviewed in [159]). Thus, polyPs are considered a less dangerous alternative to other water softeners and anticorrosion agents [159]. PolyPs are also broadly used in the food industry as a food, specifically meat, preservative, and water retaining agent. Still, taking into account the important role of polyP in cell metabolism, bone tissue development, and blood coagulation, it is also necessary to control polyP amounts in food.

Importantly, while the ability of microalgae to store P as polyP granules has been repeatedly demonstrated for decades, the exact chemistry of the granules still remains unclear. The central paradigm is that the polyP granules are formed from linear orthophosphoric acid units linked by phosphoanhydride bonds surrounded by counter-ions [70] and possibly other (organic) compounds [15,61]. While several reports showed that microalgal cells at different cellular ages stored different amounts of polyP in different intracellular locations, the knowledge about different structures of polyPs (e.g., short vs. long chain polyp, etc.), according to cell age or ecology is currently elusive. Because different types of polyP have specific chemical characteristics and consequently different biotechnological applications, polyP characterization should be an area of future investigation in the field of microalgal P up-cycling.

## 4. Conclusions and Outlook

Sustainable usage of P, an irreplaceable macronutrient, is essential to solve the pressing problem, i.e., to ensure food security for the increasing population while preserving fragile ecosystem services and biodiversity. This problem is exacerbated by the exhaustion of water and fossil energy supply (which are necessary for the processing of rock phosphate) as well as by geopolitical complications. Stricter P discharge regulations are expected to intensify the cooperation of wastewater treatment plants and industrial partners, also in the field of bioremediation and circular bioeconomy. According to [6], a favored strategy in Germany is mono-combustion of the sludge and purification of the P*_i_* from the residual ash which can be, in principle, accomplished with microalgal cultures.

This review clearly shows that microalgae have a formidable potential for valorizing P-rich waste to increase the sustainability of the usage of this indispensable macronutrient. The scientific community has made significant progress in understanding the interactions in the P microalgae–plant–environment system. Such advances have improved our understanding of the mechanisms of P acquisition, allocation, and regulation thereof at the molecular, cellular, organism, and community levels. Still, current knowledge of P metabolism and its regulation in microalgae is insufficient for the development of a robust, economically viable technology harnessing microalgae for efficient capture of P from waste and turning it into valuable products. Particularly promising are the biofertilizers from P-rich microalgal biomass helping to re-route P from the anthropogenic output to agricultural ecosystems. Thus, the evidence on the interplay between native soil microbiome and the microbiome of microalgal culture introduced to the soil with microalgal biomass remains scarce and often limited to phenomenological descriptions.

P biofertilizers from microalgal biomass will especially be beneficial and hence most efficient when applied to marginal soils and soils low on organic carbon content. Further effort is required to identify the strains combining a high P biocapture efficiency with resilience to wastewater components and fast growth capability. Native and artificial consortia formed by diverse microbes around microalgae deserve close attention in this regard for their synergistic beneficial effects on the soil and crop plants. More field tests including different crops, soil types, and agronomic practices needed to evaluate the agronomic efficiency of the microalgae-based P biofertilizers. 

A basic problem is finding the most economically efficient cultivation system and cutting down on microalgal biomass production costs. Most likely it will be an outdoor growing system leveraging local waste nutrient sources (waste streams and concentrated CO_2_, e.g., from flue gas). To make sure that the developed solutions for P recycling with microalgae are economically feasible, a detailed techno-economic analysis will be required along with the assessment of greenhouse emissions and secondary environmental impacts. Further steps in this direction can be made by bioprospecting microalgal strains more efficiently at P*_i_* uptake and polyP accumulation [36]. 

The biotechnological achievements should complement other essential advances such as crop varieties bred for the P use efficiency, precision P fertilizer applications, and other measures needed to cut down the P loss to groundwater, inland water systems, and the ocean with a corresponding decrease in eutrophication. Further study of polyP biochemistry and cell biology is important to expand their applications in medicine, environmental protection, and agriculture. The problem of research standardization and the need for strain benchmarking are highly relevant to the progress in this field.

A critical breakthrough in sustainable usage of P must be achieved to slow down our linear P*_i_* use—from the mining of phosphate rock to human P*_i_* applications and finally to P sedimentation in the ocean (as highlighted by Blank [6] and Raven [161]) before the available phosphate reserves will be exhausted. This is projected to take place within two centuries or even earlier. Prudent use of the currently available and novel knowledge and technologies inspires us with hope to significantly delay—if not avoid—a looming P crisis, and to mitigate environmental and geopolitical problems associated with sustainable P use.

## Figures and Tables

**Table 1:** Ranges of P and/or P*_i_* concentrations in pristine natural and anthropogenically impacted habitats and typical organisms.

Habitat or Source	P or P*_i_* Range	Ref.
Wastewater	3–330 mg L^−1^ (3 µM–3 mM) P*_i_*	[48]
	Domestic: 0.5–8.6 mg-P L^−1^ (0.02–0.3 mM)	[49]
	Industrial, e.g., mine drainage: 186–558 mg-P L^−1^ (6–18 mM)	[50]
Deep aphotic ocean	Soluble reactive P: 0.8–5.4 µg-P L^−1^ (0.025–0.175 µM)	[51]
River	Total P 0.04–0.4 mg-P L^−1^ (0.001–0.01 mM)	[52]
Lakes	Soluble reactive P: 0.01–0.85 mg-P^−1^ (0.3 µM–0.3 mM))	[53]
Unfertilized soil	12.4–341 µg-P L^−1^ (0.4–−11 μM) *	[2]
Fertilized soil	0.1–5.0 mg-P L^−1^ (0.003–0.16 mM)	[54]
Intracellular concentration	Crop plants: 155–620 mg-P L^−1^ (5–20 µM)	[55]
	Microalgae: *C. reinhardtii*: 152 ± 37.0 µg free P*_i_* (4.9 ± 1.2 µmol mostly in the chloroplast)	[56]

* Bioavailable for plants from the soil solution.

## Supplementary Materials

The following supporting information can be downloaded at: https://www.mdpi.com/article/10.3390/plants13131834/s1, Table S1: The key Pi transporters of terrestrial plants; Table S2: Key genes involved in P acquisition and polyP metabolism in eukaryotic microalgae with *C. reinhardtii* as reference. Most of the genes involved are under control of the transcription factor psr1 (see further detail in [162,163,164]); Table S3: Key genes involved in P acquisition and polyP metabolism in cyanobacteria with *Synechococcus* sp. as reference. In prokaryotes, the genes constituting the pho regulon are controlled by the transcription factor PhoB. Based on the Pho regulon components of *Synechococcus* sp. WH8102 described here [103], we provided available data for *Synechococcus* sp. WH8102 as well as *Synechococcus* sp. strain WH7803 using Uniprot database. References [162,163,164,165,166,167,168,169] are included in the supplementary materials.
