# Supplementary material for: Getting Grip on Phosphorus: Potential of Microalgae as a Vehicle for Sustainable Usage of This Macronutrient
Source: Plants (Basel). 2024 Jul 3;13(13):1834. doi: 10.3390/plants13131834 (PMC11243885; doi:10.3390/plants13131834)
Supplement: Supplementary file 1 [file plants-13-01834-s001.zip › plants-3040980-supplementary.pdf]

Table S1. The key P_i transporters of terrestrial plants.

Transporter	Organism	Remarks	Refs.
*PTx family of high-affinity P_i transporters (e.g. AtPT1 from *A. thaliana*)	*A. thaliana*, *Licopersicon esculentum*, *Medicago sativa*, *Nicotiana tabacum* etc.	Membrane proteins from MFC family.	[1-3]
Phtx family of low-affinity P_i transporters	*A. thaliana*, *Oryza sativa* etc.	Pi:H+ symporters; apparent K_m for P_i — 0.4 mM	
PHR1, homolog of Psr1	*A. thaliana*, *Brassica* spp. etc.	P_i starvation-inducible, homologous to β-glucosidase, may induce deglycosylation and regulation of acid phosphatases during P_i starvation	

Table S2. Key genes involved in P acquisition and polyP metabolism in eukaryotic microalgae with C. *reinhardtii* as reference. Most of the genes involved are under control of the transcription factor psr1 (see further detail in [4-6])

Gene or enzyme	Annotation 1	Annotation 2	Remarks	Refs.
Psr1 (phosphorus starvation regulator 1)	(Cre12.g495100)	Q9S807	Putative transcriptional activator, crucial for acclimation of the alga to P_i starvation	[5,7]
Lpb1 (low-P bleaching)	(Cre12.g554250)	Q4VGM5		[8]
Pta1-4	(Cre02.g075050; Cre16.g686800; Cre16.g686750; Cre16.g686850)	Q8LP71 Q8LP70 Q8LP69 A8ISD7	P_i transporter (H^+/P_i family)	
Ptb1–5,6a,7–9,12	Cre12.g491600, Cre07.g325741, Cre07.g325740, Cre02.g144750, Cre02.g144700, Cre16.g655200, Cre12.g489400, Cre16.g676757, Cre02.g144600, Cre02.g144650)	Q8LP68 Q8LP67 A8JH07 A0A2K3E3U8 A8J0U2 A8J994 A0A2K3D267 - A0A2K3E3X0 A8J0U1	P_i transporter (Na^+/P_i family)	
Ptc1	(Cre06.g251650)	A0A2K3DLZ3	P_i transporter (low affinity)	
Phox	(Cre04.g216700)	A0A2K3DTA4	Calcium-dependent alkaline phosphatase	
Pho1	(Cre08.g359300)	A8JGF3	Alkaline phosphatase	
Phod	(Cre05.g239850)	A0A2K3DT40	Alkaline phosphatase	
Mpa1,2,8,9,11,13	Cre03.g146207, Cre12.g500250,	A8J2X5 -	Calcineurin-like phosphatase	

Gene or enzyme	Annotation 1	Annotation 2	Remarks	Refs.
	Cre11.g468500,<br>Cre11.g476700,<br>Cre13.g578350,<br>Cre16.g672250)	A0A2K3D861<br>-<br>-<br>A0A2K3CVM3		
Vtc1	(Cre12.g510250)	A8IKM0	Vacuolar transporter chaperone family (putative polyP synthesis)	
Vtc4	Cre09.g402775	A0A2K3DF66		
Vtc$_x$	(Cre01.g005500,<br>Cre10.g461500,<br>Cre10.g461500,<br>Cre09.g402812)	A0A2K3E503<br>A0A2K3DBW4<br>A0A2K3DF49		

Table S3. Key genes involved in P acquisition and polyP metabolism in cyanobacteria with *Synechococcus sp* as reference. In prokaryotes, the genes constituting the pho regulon are controlled by the transcription factor PhoB. Based on the Pho regulon components of *Synechococcus* sp. WH8102 described here [9], we provided available data for *Synechococcus* sp. WH8102 as well as *Synechococcus* sp. strain WH7803 using Uniprot database.

Gene/ enzyme	Annotation 1	Annotation 2	Remarks	Ref
phoR	SynWH7803_1546	Q56181	Activated during P depletion, a regulatory component that phosphorylates phoB. Also annotated as sphS in other Synechococcus species (P39664, for S elongatus)	[10]
phoB	SynWH7803_1545	Q56180	Controls transcription of the Pho regulon. Also annotated as sphR in other Synechococcus species (P39663, for S elongatus)	
ptrA	SynWH7803_1046	A5GKK7	Protein with a potential regulatory role under P depletion/stress	
phoU	Syn7502_00586	K9SQ49	A regulatory protein regulating Pi import through the Pst system by interacting with PstB and PhoB	[11]
pstC	SynWH7803_1245	A5GL56	Subunits constituting the Pi import complex	[12]
pstSI	SynWH7803_2513	A5GPS4		
pstSII	SynWH7803_1045	A5GKK6		
pstA	SynWH7803_1244	A5GL55		
pstB	SynWH7803_1243	A5GL54		
phnC	SynWH7803_1469	A5GLT0	Subunits consituting the phosphonate-ABC transporter	
phnD	SynWH7803_1471	A5GLT2		
phnE	SynWH7803_1470	A5GLT1		
phoA	syc0750_c	A0A0H3K146		

Gene/ enzyme	Annotation 1	Annotation 2	Remarks	Ref
phoD	SynWH7803_1802	A5GMR3	Alkaline phosphatases	
phoV		Q55320		
ppk1	SYNW2495	Q7U3D7	Polyphosphate kinase 1	[13]
ppx	SynWH7803_1855	A5GMW6	Exopolyphosphatase	

Supplementary references

1. Grossman, A.; Takahashi, H. Macronutrient utilization by photosynthetic eukaryotes and the fabric of interactions. *Annual Review of Plant Biology* **2001**, *52*, 163-210.
2. Raghothama, K. Phosphate acquisition. *Annual review of plant biology* **1999**, *50*, 665-693.
3. Raghothama, K. Phosphate transport and signaling. *Current opinion in plant biology* **2000**, *3*, 182-187.
4. Bajhaiya, A.K.; Dean, A.P.; Zeef, L.A.; Webster, R.E.; Pittman, J.K. PSR1 is a Global Transcriptional Regulator of Phosphorus Deficiency Responses and Carbon Storage Metabolism in Chlamydomonas reinhardtii. *Plant physiology* **2015**, pp. 01907.02015.
5. Wykoff, D.D.; Grossman, A.R.; Weeks, D.P.; Usuda, H.; Shimogawara, K. Psr1, a nuclear localized protein that regulates phosphorus metabolism in Chlamydomonas. *Proceedings of the National Academy of Sciences* **1999**, *96*, 15336-15341.
6. Sanz-Luque, E.; Grossman, A.R. Chapter 4 - Phosphorus and sulfur uptake, assimilation, and deprivation responses. In *The Chlamydomonas Sourcebook (Third Edition)*, Grossman, A.R., Wollman, F.-A., Eds. Academic Press: London, 2023; https://doi.org/10.1016/B978-0-12-821430-5.00006-7pp. 129-165.
7. Moseley, J.L.; Chang, C.-W.; Grossman, A.R.J.E.c. Genome-based approaches to understanding phosphorus deprivation responses and PSR1 control in *Chlamydomonas reinhardtii*. **2006**, *5*, 26-44.
8. Chang, C.W.; Moseley, J.L.; Wykoff, D.; Grossman, A.R. The LPB1 gene is important for acclimation of Chlamydomonas reinhardtii to phosphorus and sulfur deprivation. *Plant Physiol* **2005**, *138*, 319-329, doi:10.1104/pp.105.059550.
9. Tetu, S.G.; Brahamsha, B.; Johnson, D.A.; Tai, V.; Phillippy, K.; Palenik, B.; Paulsen, I.T. Microarray analysis of phosphate regulation in the marine cyanobacterium Synechococcus sp. WH8102. *The ISME Journal* **2009**, *3*, 835-849.
10. Dyhrman, S.T. Nutrients and Their Acquisition: Phosphorus Physiology in Microalgae. In *The Physiology of Microalgae*, Borowitzka, M.A., Beardall, J., Raven, J.A., Eds. Springer International Publishing: Cham, 2016; 10.1007/978-3-319-24945-2_8pp. 155-183.
11. Hudek, L.; Premachandra, D.; Webster, W.A.; Bräu, L. Role of Phosphate Transport System Component PstB1 in Phosphate Internalization by Nostoc punctiforme. *Appl Environ Microbiol* **2016**, *82*, 6344-6356, doi:10.1128/aem.01336-16.
12. Tiwari, B. Chapter 7 - Phosphate metabolism in cyanobacteria: fundamental prospective and applications. In *Cyanobacteria*, Mishra, A.K., Singh, S.S., Eds. Academic Press: 2024; https://doi.org/10.1016/B978-0-443-13231-5.00002-7pp. 159-175.
13. Gomez-Garcia, M.R.; Fazeli, F.; Grote, A.; Grossman, A.R.; Bhaya, D.J.J.o.b. Role of polyphosphate in thermophilic Synechococcus sp. from microbial mats. **2013**, *195*, 3309-3319.
